# Chemical Composition and Antimicrobial Activity of the Essential Oils from Two Species of *Thymus* Growing Wild in Southern Italy

**DOI:** 10.3390/molecules14114614

**Published:** 2009-11-12

**Authors:** Laura De Martino, Maurizio Bruno, Carmen Formisano, Vincenzo De Feo, Francesco Napolitano, Sergio Rosselli, Felice Senatore

**Affiliations:** 1 Dipartimento di Scienze Farmaceutiche, Università degli Studi di Salerno, via Ponte don Melillo, 84084 Fisciano (Salerno), Italy; E-Mail: defeo@unisa.it (V.D.F); 2 Dipartimento di Chimica Organica, Università di Palermo, Viale delle Scienze, Parco d’Orleans II, 90128 Palermo, Italy; E-Mails: bruno@dicpm.unipa.it (M.B.); rosselli@unipa.it (S.R.); 3 Dipartimento di Chimica delle Sostanze Naturali, Università degli Studi di Napoli, “Federico II”, Via D. Montesano, 49, 80131 Napoli, Italy; E-Mails: caformis@unina.it (C.F.); fnapolitano@virgilio.it (F.N.); fesenato@unina.it (F.S.)

**Keywords:** *Thymus longicaulis* C. Presl, *Thymus pulegioides* L., essential oil composition, thymol, geraniol, antibacterial activity

## Abstract

The volatile constituents of the aerial parts of two samples of *Thymus longicaulis* C. Presl, collected in Campania and in Sicily, and two samples of *Thymus pulegioides* L. from the same regions, were extracted by hydrodistillation and analyzed. Considering the four oils together, seventy-eight different compounds were identified: 57 for *Thymus longicaulis* from Sicily (91.1% of the total oil), 40 for *Thymus longicaulis* from Campania (91.5% of the oil), 39 for *Thymus pulegioides* from Sicily (92.5% of the oil) and 29 for *Thymus pulegioides* from Campania (90.1% of the oil). The composition of the oils is different, although the most abundant components are identical in *T. pulegioides*. The essential oils showed antibacterial activity against eight selected microorganisms.

## 1. Introduction

Among the aromatic plants belonging to the Lamiaceae family, the genus *Thymus* is noteworthy for the numerous species and varieties of wild-growing plants. This genus comprises about 400 species of perennial aromatic, evergreen or semi-evergreen herbaceous plants with many subspecies, varieties, subvarieties and forms; the species are endemic and widely distributed, growing in the temperate and cold regions of the Old World, being native to Southern Europe and diffused in particular in the Mediterranean area. The plants are extensively used, fresh and dried, as a culinary herb. Their essential oils are utilized as flavour ingredients in a wide variety of food, beverage and confectionery products, as well as in perfumery for the scenting of soaps and lotions. Because of their antiseptic, antispasmodic and antimicrobial properties, they are also used for medicinal purposes [1,2,3,4,5].

In recent years, several reports have been published concerning the composition and/or the biological properties of *Thymus* essential oils. These studies have emphasized the existence of marked chemical differences among oils extracted from different species or varieties. More than 20 essential oil chemotypes were noticed in different species of *Thymus* genus [6], but, on the other hand, different chemotypes can grow in the same habitat, so the study of this genus could be interesting [7]. This chemical diversity can influence the biological activity of the oils and it is generally a function of three factors: genetical, physiological conditions and the environment [8]. Moreover, most aspects of medicinal use of *Thymus* spp. are related to their essential oil composition, which shows various levels of thymol and/or carvacrol, phenolic derivatives with strong and wide-spectrum antimicrobial activity [5,8].

*T. longicaulis* C. Presl is a species with long, somewhat woody, creeping branches, non flowering or with a terminal inflorescence [9]. This species is important from the ethnobotanical point of view as a traditional medicinal plant [10,11]: it is reported as an antiseptic, an expectorant and a spasmolytic, properties probably correlated with the content of essential oils and flavonoids. The composition of essential oil from *T. longicaulis* has been studied previously [12,13,14,15,16].

Eight chemotypes were determined for *Thymus pulegioides* L. [6,17]. This species is widely distributed in the European continent and in Mediterranean islands. In Portugal, it grows in the northeast, and it is locally used as an antiseptic. Previous results have demonstrated that this species is polymorphic [18], and that the thymol/carvacrol chemotype is one of the most abundant in Portugal. In Italy, it is traditionally used as an expectorant, an anthelmintic, a gastric antispasmodic, and an astringent [19].

As a continuation of our research on the oils of the Lamiaceae growing wild in Southern Italy [20,21] and because of this variability of *Thymus* genus, we examine in this work the composition of the essential oils of *Thymus longicaulis* and *Thymus pulegioides* growing wild in two different regions of Southern Italy (Campania and Sicily) and their antimicrobial activity on eight selected microorganism.

## 2. Results and Discussion

### 2.1. Chemical composition of the essential oils

Considering the four oils together a total of seventy-eight different compounds were identified: 57 for *T. longicaulis* from Sicily (91.1% of the total oil), 40 for *T. longicaulis* from Campania (91.5% of the oil), 39 for *T. pulegioides* from Sicily (92.5% of the oil) and 29 for *T. pulegioides* from Campania (90.1% of the oil). The components are listed in Table 1, classified in eight classes on the basis of their chemical structures.

**Table 1:** Essential oil composition (% of total) of aerial parts of *Thymus longicaulis* C. Presl from Sicily (*Tls*) and from Campania (*Tlc*), *Thymus pulegioides* L. from Sicily (*Tps*) and from Campania (*Tpc*).

Component	LRI^a^	LRI ^b^	*Tls* *%*	*Tlc*%	*Tps*%	*Tpc*%	Identification ^c^
**Monoterpene hydrocarbons**			**25.6**	**1.8**	**30.2**	**25.2**	
α-Thujene	925	1035	0.6	t	1.2	0.8	1,2
α-Pinene	938	1075	1.3	t	1.1	1.4	1,2,3
Camphene	953	1076		t		0.5	1,2,3
Sabinene	973	1132	0.1		0.1		1,2
β-Pinene	978	1118	0.4		0.3		1,2,3
Myrcene	993	1173	4.2		0.7	0.9	1,2,3
α-Phellandrene	1005	1150	0.1		0.1		1,2,3
δ^3^-Carene	1010				0.1		1,2
α-Terpinene	1013	1189	0.8		1.4	0.9	1,2
*o-*Cymene	1020	1187	t				1,2
*p*-Cymene	1025	*1278*	9.0	0.4	17.6	19.9	1,2,3
Limonene	1030	1205	3.4	0.5	1.8	0.8	1,2,3
(*Z*)-β-Ocimene	1038	1243		t			1,2,3
(*E*)-β-Ocimene	1049	1262	0.1	t			1,2
γ-Terpinene	1057	1256	5.5	0.9	5.7		1,2
Terpinolene	1086	1265	0.1		0.1		1,2,3
**Oxygenated monoterpenes**			**8.2**	**44.8**	**12.2**	**9.4**	
1,8-Cineole	1034	1213	1.2	t	1.0	0.7	1,2,3
*cis*-Linalool oxide (furanoid)	1076	1477	0.1				1,2
*trans*-Linalool oxide (furanoid)	1087	1451	0.3				1,2
*trans*-Sabinene hydrate	1093	1474	0.5	0.5	0.4		1,2
Linalool	1097	1553	3.5	0.6	5.6	4.7	1,2,3
Camphor	1145	1532	1.3	0.6	1.0	t	1,2,3
Borneol	1167	1718	0.5	3.8	2.8	1.9	1,2,3
Terpinen-4-ol	1176	1611	0.8	0.4	0.8	2.1	1,2,3
*p*-Cymen-8-ol	1185	1856		1.2	0.6	t	1,2
α-Terpineol	1189	1706	0.1				1,2,3
Nerol	1226	1809		1.4			1,2,3
Geraniol	1235	1857		34.2			1,2,3
Neral	1237			0.4			1,2
Geranial	1267			0.7			1,2
Cumin alcohol	1288	2112		1.0			1,2
**Sesquiterpene hydrocarbons**			**20.0**	**4.8**	**9.9**	**11.1**	
Bicycloelemene	1339	1494		t			1,2
α-Cubebene	1348	1466			t		1,2
α-Copaene	1377	1497	0.4	0.3			1,2
β-Cubebene	1382	1549	0.1				1,2
β-Bourbonene	1385	1558	0.7			t	1,2
β-Elemene	1387	1600	0.1				1,2
Longifolene	1403			0.1			1,2
β-Caryophyllene	1415	1612	5.7	2.2	5.9	7.5	1,2,3
β-Gurjunene	1432	1612	0.4		0.1		1,2
γ-Elemene	1432	1650			t	t	1,2
Acoradiene	1442		0.1				1,2
(*Z*)-β-Farnesene	1443	1668	1.3				1,2
(*E*)-β-Farnesene	1452	1672	0.7				1,2
α-Humulene	1455	1689	0.4	t	0.3	1.2	1,2
*allo*-Aromadendrene	1463	1662	0.2	t			1,2
Germacrene D	1477	1726	5.3		0.4	0.2	1,2
γ-Muurolene	1478	1704	t	0.5	0.2		1,2
Viridiflorene	1494	1687	0.1		0.2		1,2
Valencene	1494	1740	0.1	0.1			1,2
α-Muurolene	1503	1740	0.2	0.1			1,2
β-Bisabolene	1510	1743	0.5	1.5	2.6	0.9	1,2
γ-Cadinene	1515	1776	1.3				1,2
δ-Cadinene	1526	1773			0.2	1.3	1,2
α-Cadinene	1535		0.2				1,2
Germacrene B	1554	1854	2.2				1,2
**Oxygenated sesquiterpenes**			**10.1**	**0.4**	**2.3**	**2.2**	
Caryophyllene oxide	1579	2008			1.9	2.1	1,2,3
Guaiol	1598	2108	0.2				1,2
t-Cadinol	1640	2158	9.2	0.3			1,2
t-Muurolol	1642	2209	0.1				1,2
α-Cadinol	1652	2255	0.4	0.1			1,2
(*Z,Z*)-Farnesol	1715		0.1		0.2	0.1	1,2
14-Hydroxy-α-humulene	1718		0.1				1,2
(*Z*)-Lanceol	1765				0.2		1,2
**Phenolic compounds**			**26.4**	**38.7**	**36.4**	**39.6**	
Thymol methyl ether	1239	1607	5.5	0.8	10.8	6.0	1,2
Carvacrol methyl ether	1245	1975		0.5		2.2	1,2
Thymol	1293	2198	6.4	9.3	21.8	26.3	1,2,3
Carvacrol	1299	2239	1.7	14.5	3.1	4.7	1,2,3
Thymyl acetate	1365	1865	12.8		0.7	0.4	1,2
Carvacryl acetate	1367	1885		13.6			1,2
**Hydrocarbons**				**1.0**			
Dodecane	1200	1200		0.6			1,2,3
Tridecane	1300	1300		0.4			1,2,3
**Fatty acids**						**0.6**	
Hexadecanoic acid	1957	2931	t	t		0.6	1,2,3
**Others**			**0.8**		**1.5**	**2.0**	
1-Octen-3-ol	980	1454	0.6		1.3	2.0	1,2
3-Octanol	992	1394			0.1		1,2,3
Octanol	1073	1562	0.1		0.1		1,2,3
Nonanol	1170		0.1				1,2
**Total amount of compounds**			**91.1**	**91.5**	**92.5**	**90.1**	

^a^: HP-5MS column; ^b^: HP Innowax column; ^c^: 1 = LRI linear retention index; 2 = MS identification based on comparison of mass spectra; 3 = Co-GC retention time identical to authentic compounds.

For *T. longicaulis* from Sicily, the monoterpene and sesquiterpene fractions were present in quite similar amounts, 33.8% and 30.1%, respectively. The monoterpene fraction was almost entirely constituted by monoterpene hydrocarbons (25.6%), among which the main representatives were *p*-cymene (9.0%), γ-terpinene (5.5%), myrcene (4.2%) and limonene (3.4%). Among oxygen containing monoterpenes, linalool (3.5%), camphor (1.3%) and 1,8-cineole (1.2%) were the most abundant. τ-Cadinol (9.2%) was the main component of the sesquiterpene fraction of the oil; other abundant constituents of this fraction were β-caryophyllene (5.7%), germacrene D (5.3%) and germacrene B (2.2%). Another abundant fraction was constituted by phenolic compounds (26.4%) with a prevalence of thymyl acetate (12.8%) followed by thymol (6.4%) and thymol methyl ether (5.5%).

The oil from *T. longicaulis* collected in Campania presented a different composition. In this oil monoterpenes also constituted the most abundant fraction (46.6%), but with a prevalence of oxygen containing monoterpenes (44.8%) with a great amount of geraniol (34.2%) and borneol (3.8%). The phenols carvacrol (14.5%), carvacryl acetate (13.6%) and thymol (9.3%) were also present in a significant percentages. Among sesquiterpenes, only β-caryophyllene (2.2%) was present in appreciable amounts.

The two studied oils, even if characterized by a prevalence of monoterpenes, are quite different in the relative percentages of monoterpene hydrocarbons and oxygenated monoterpenes: the former are predominant in *T. longicaulis* collected in Sicily, while the oxygenated monoterpenes were only 8.2%. Moreover, in the *T. longicaulis* from Campania, the predominant compound is geraniol, which is absent in the other oil. Our data disagree with those previously reported for *T. longicaulis* collected in Mid-Western Turkey [22] in which thymol is the main component. Baser *et al.* [13,14,15] also reported thymol as predominant components of different samples of this species. On the other hand, the composition of three chemotypes of *T. longicaulis* subsp. *chaubardii* from Turkey again, studied by Tzakou [23], shows the main components were oxygenated monoterpenes (geraniol, linalool and geranyl acetate). These differences in the essential oils of *Thymus longicaulis* confirmed that the genus *Thymus* is taxonomically and genetically complex [19].

Monoterpenes constituted the most abundant fraction of *Thymus pulegioides* from Sicily (42.4%), with a prevalence of monoterpene hydrocarbons (30.2%) among which *p*-cymene (17.6%) and γ-terpinene (5.7%) predominated. Among seven oxygen containing monoterpenes (12.2%), linalool (5.6%) and borneol (2.8%) were the most abundant. Ten sesquiterpene hydrocarbons (with two compounds in trace amounts) accounted for the 9.9% of the total oil, with β-caryophyllene (5.9%) as main compound, while caryophyllene oxide (1.9%) was the only abundant oxygenated sesquiterpene. Thymol (21.8 %) and thymol methyl ether (10.8 %) represented the main components of the phenolic fraction (36.4%).

The essential oil of *Thymus pulegioides* from Campania was mainly constituted by phenolic compounds (39.6%), being thymol (26.3%) the main one. The monoterpene fraction (34.6%) comprised mainly hydrocarbons (25.2%) with *p*-cymene (19.9%) as predominant component. Among oxygen containing monoterpenes, linalool (4.7%), terpinen-4-ol (2.1%) and borneol (1.9%) were the most abundant compounds. β-Caryophyllene (7.5%) represented the main sesquiterpene hydrocarbon.

Moreover, the two samples studied of *T. pulegioides* have a similar composition pattern and both belong to a thymol chemotype. Our results are in agreement with previous data on the essential oils of the Italian wild growing *T. pulegioides*, which were defined as belonging to the thymol chemotype according to the high content of thymol, *p*-cymene, and γ-terpinene [19]. On the other hand, previous papers on the essential oils of different varieties of *T. pulegioides,* concluded that there is no clear chemical relation between the varieties and chemotypes [24].

### 2.2. Antimicrobial activity

The *in vitro* antibacterial activity of *Thymus longicaulis* and *T. pulegioides* against eight bacterial species was evaluated by the *in vitro* paper-disk diffusion method [25]. The microorganism selected are representative of the Gram positive and Gram negative classes and known to cause respiratory, gastrointestinal, skin and urinary disorders in humans. The results obtained in the antibacterial assay are shown in Table 2a and Table 2b. Our samples were more active against Gram positive bacteria but generally are less active than gentamycin and tetracyclin. This finding totally agrees with the observations derived from studies with essential oils from other thyme species. Nedorostova *et al.* [26] reported, in particular, the antimicrobial activity in vapour phase of essential oils of *Thymus pulegioides*; instead, Ložiene *et al.* [27] reported the antibacterial activity of extracts of this species.

**Table 2a:** Antibacterial activity (diffusion method) of different amounts of the essential oil of *T. longicaulis*. Inhibition is expressed in mm and include the diameter of paper disc (6 mm). Results are shown as mean ± standard deviation (SD) of the inhibition zone (n = 3).

	*Thymus longicaulis* from Sicily					*Thymus longicaulis* from Campania					G^a^	T^b^
	Concentration tested (mg/ml)					Concentration tested (mg/ml)						
**Bacteria **	**10**	**5**	**2.55**	**1.25**	**0.62**	**10**	**5**	**2.55**	**1.25**	**0.62**		
*S. aureus*	21 ± 1.3	18 ± 0.3	13 ± 1.1	10 ± 1.0	7 ± 0.8	18 ± 1.1	15 ± 1.2	10 ± 0.3	8 ± 1.1	n.a.	29 ± 1.4	n.t
*S. faecalis*	18 ± 0.9	14 ± 1.3	10 ± 1.0	7 ± 0.3	n.a.	15 ± 0.9	12 ± 1.3	8 ± 0.6	n.a	n.a	30 ± 1.0	n.t
*B. subtilis*	15 ± 1.0	12 ± 0.8	9 ± 0.6	n.a	n.a	11 ± 1.0	7 ± 1.0	n.a	n.a	n.a	28 ± 1.2	n.t
*B. cereus*	16 ± 0.3	13 ± 1.1	10 ± 0.3	7 ± 0.9	n.a	14 ± 1.9	10 ± 0.3	7 ± 0.4	n.a	n.a	28 ± 1.3	n.t
*P. mirabilis*	12 ± 1.0	9 ± 0.7	n.a	n.a	n.a	9 ± 1.8	7 ± 0.4	n.a	n.a	n.a	n.t	29 ± 1.1
*E. coli*	17 ± 0.6	13 ± 1.0	10 ± 0.2	7 ± 0.8	n.a	14 ± 0.3	10 ± 0.5	7 ± 0.7	n.a	n.a	n.t	26 ± 1.5
*S. typhi Ty2*	13 ± 0.3	10 ± 1.1	7 ± 0.4	n.a	n.a	11 ± 1.2	10 ± 1.2	7 ± 0.8	n.a	n.a	n.t	21 ± 0.9
*P. aeruginosa*	15 ± 0.0	11 ± 1.2	8 ± 0.9	n.a	n.a	13 ± 1.4	9 ± 1.5	n.a	n.a	n.a	n.t	28 ± 1.3

^a^ Gentamycin (10 μg); ^b^ Tetracyclin (15 μg), n.a.= not active; n.t.= not tested.

**Table 2b:** Antibacterial activity (diffusion method) of different amounts of the essential oil of *T. pulegioides*. Inhibition is expressed in mm and include the diameter of paper disc (6 mm). Results are shown as mean ± standard deviation (SD) of the inhibition zone (n = 3).

	Thymus pulegioides from Sicily					Thymus pulegioides from Campania					G^a^	T^b^
	Concentration tested (mg/ml)					Concentration tested (mg/ml)						
**Bacteria**	**10**	**5**	**2.55**	**1.25**	**0.62**	**10**	**5**	**2.55**	**1.25**	**0.62**		
*S. aureus*	17 ± 1.0	14 ± 1.2	11 ± 1.1	8 ± 1.0	n.a	20 ± 0.9	17 ± 1.1	14 ± 1.2	19 ± 1.3	7 ± 0.9	29 ± 1.4	n.t
*S. faecalis*	16 ± 0.3	13 ± 1.4	10 ± 1.2	7 ± 0.9	n.a.	16 ± 1.3	13 ± 1.2	9 ± 1.3	7 ± 1.2	n.a	30 ± 1.0	n.t
*B. subtilis*	12 ± 0.9	9 ± 1.5	n.a.	n.a.	n.a.	16 ± 1.2	13 ± 1.4	10 ± 1.0	7 ± 1.0	n.a	28 ± 1.2	n.t
*B. cereus*	15 ± 1.2	11 ± 1.6	8 ± 1.0	n.a.	n.a	15 ± 1.4	11 ± 1.1	8 ± 0.3	n.a	n.a	28 ± 1.3	n.t
*P. mirabilis*	11 ± 1.5	7 ± 1.2	n.a.	n.a	n.a	11 ± 1.1	7 ± 0.5	n.a	n.a	n.a	n.t	29 ± 1.1
*E. coli*	15 ± 1.4	12 ± 1.4	9 ± 1.4	7 ± 0.7	n.a	16 ± 1.2	12 ± 1.1	9 ± 0.6	7 ± 1.1	n.a	n.t	26 ± 1.5
*S. typhi Ty2*	13 ± 1.2	9 ± 1.0	n.a.	n.a	n.a	12 ± 1.1	8 ± 1.3	n.a	n.a	n.a	n.t	21 ± 0.9
*P. aeruginosa*	13 ± 1.1	10 ± 1.3	7 ± 0.9	n.a.	n.a	13 ± 1.5	10 ± 1.5	7 ± 0.5	n.a	n.a	n.t	28 ± 1.3

^a^ Gentamycin (10 μg); ^b^ Tetracyclin (15 μg), n.a.= not active; n.t.= not tested.

Our result agree with the study of *Thymus longicaulis* L. of Chorianopoulos and coworkers [11] who reported the antimicrobial activity of this species’ essential oil against five common foodborne pathogens. Generally, the chemical composition of the oils determine their antimicrobial activity; Valero and Giner reported the effects of antimicrobial activity of essential oil components, particularly of phenols, such as carvacrol and thymol [28]. The antimicrobial activity of *Thymus* species and *Thymus pulegioides*, in particular, was attributed to the presence of thymol [29,30]; in reality, even if antimicrobial activity of an essential oil is often attributed mainly to its major components, today it is known that the synergistic or antagonistic effect of one compound in minor percentage of mixture has to be considered [31].

## 3. Experimental Section

### 3.1. Plant material

Aerial parts of *Thymus longicaulis* were collected, at the full flowering stage in Sicily at Madonie a high mountain group of Palermo province, and in Campania, near the town of Agerola, Naples province; aerial parts of *Thymus pulegioides* L. were collected at the full flowering stage, at Madonie and near Agerola. Plants were identified by the Prof. V. De Feo. Voucher specimens of the plants are deposited in the Herbarium of the Medical Botany Chair, at the University of Salerno.

### 3.2. Isolation of the volatile components

The air-dried samples (50 g) were ground in a Waring blender and then subjected to hydrodistillation for 3 h using *n*-hexane as a solvent, according to the standard procedure previously described [21]. The extracts were dried over anhydrous sodium sulphate and then stored in sealed vials, at -20 °C, ready for the GC and GC-MS analyses. The samples yielded 0.8% (v/w) *Thymus longicaulis* from Campania, 0.7% (v/w)*T. longicaulis* from Sicily, 0.9% (v/w) *T. pulegioides* from Campania and 1.1% (v/w) *T. pulegioides* from Sicily as yellow oils, with a pleasant smell.

### 3.3. GC and GC/MS analyses

GC analyses were carried out on a Hewlett Packard Sigma 115 gas chromatograph equipped with a FID and a 30 m × 0.25 mm i.d.. HP 5MS fused silica capillary column (film thickness: 0.25 μm). Column temperature: 40 °C, with 5 min initial hold, and then to 270 °C at 2 °C/min, 260 °C (20 min); injection mode splitless (1 µL of a 1:1,000 *n*-pentane solution). Injector and detector temperatures were 250 °C and 290 °C, respectively. Analysis was also run by using a fused silica HP Innowax polyethylenglycol capillary column (50 m × 0.20 mm, 0.25 µm film thickness). In both cases carrier gas was He, with flow rate of 1 mL/min. GC-MS analyses were performed. Analysis on an Agilent 6850 Ser. II apparatus, fitted with a fused silica DB-5 capillary column (30 m × 0.25 mm i.d.; 0.33 μm film thickness), coupled to an Agilent Mass Selective Detector MSD 5973; ionization energy 70 eV; electron multiplier voltage 2000 V. Mass spectra were scanned in the range 40-500 amu, scan time 5 scans/s. Gas chromatographic conditions were as reported above; transfer line temperature, 295 °C.

### 3.4. Identification of components

Most constituents were identified by gas chromatography by comparison of their linear retention indices (LRi) with either those of the literature [32,33] or with those of authentic compounds available in our laboratories. The linear retention indices were determined in relation to a homologous series of *n*-alkanes (C_8_-C_28_) under the same operating conditions. Further identification was made by comparison of their mass spectra on both columns with either those stored in NIST 02 and Wiley 275 libraries or with mass spectra from the literature [32,34] and a home-made library. Components relative concentrations were obtained by peak area normalization. No response factors were calculated.

### 3.5. Antimicrobial activity

The antimicrobial activity of the oil was evaluated by the *in vitro* paper-disk diffusion method [25] against eight selected Gram+ and Gram– bacteria: *Staphylococcus aureus* (ATTC 25923), *Streptococcus faecalis* (ATTC 29212), *Bacillus subtilis* (ATCC 6633), *Bacillus cereus* (PCI 213), *Proteus mirabilis* (ATCC 12453), *Escherichia coli* (ATCC 25922), * Salmonella typhi Ty2* (ATCC 19430), *Pseudomonas aeruginosa* (ATCC 27853). Aliquots of each oil were dissolved in dimethyl sulfoxide to give solutions containing from 10 to 0.62 mg/mL. Sterile 6 mm discs (Whatman N.1) were impregnated with 20 μL of each solution and placed on the surface of agar plates so prepared: 25 mL of Mueller-Hinton agar medium [NCCLS, 1984] and a standardized inoculum of the correspondent test microorganism (standard 0.5 McFarland). Plates were incubated at 37 °C for 24 h. The analyses were carried out in triplicate and the results are expressed as mean ± SD. Control disks with 10 μL of dimethyl sulfoxide showed no inhibition in a preliminary test. Gentamycin (10 μg) and Tetraciclyn (15 μg) served as positive control on Gram– and Gram+ bacteria, respectively.

## 4. Conclusions

Considering the remarkable variations reported for the essential oil of *T. pulegioides* from different localities, it was of interest to continue research on its chemical polymorphism. Our data reveals that the essential oils studied, from two different localities, belong to a thymol chemotype with an high percentage of *p*-cymene. The two studied oils of *Thymus longicaulis*, even if characterized by a prevalence of the monoterpene fraction, are quite different with regards to the relative percentages of monoterpene hydrocarbons and oxygenated monoterpenes and the data obtained serve to highlight the chemical polymorphism of *T. longicaulis*. Essential oils are extensively used as flavour ingredient in a wide variety of food, beverage and confectionary products. This use and the antibacterial activity of these substances make them potential natural preservatives in food industry.

## References

[1] El-Hela A.A.A. (2007). Chemical composition and biological studies of the essential oil of *Thymus decussatus* Benth growing in Egypt. Egypt. J. Biomed. Sci..

[2] Jirovetz L., Wlcek K., Buchbauer G., Gochev V., Girova T., Stoyanova A., Schmidt E. (2007). Antifungal activity of various Lamiaceae essential oils rich in thymol and carvacrol against clinical isolates of pathogenic *Candida* species. Int. J. Essent. Oil Therap..

[3] Maksimović Z., Milenković M., Vučićević D., Ristić M. (2008). Chemical composition and antimicrobial activity of *Thymus pannonicus* All. (Lamiaceae) essential oil. Cent. Eur. J. Biol..

[4] Safaei-Ghomi J., Ebrahimabadi A.H., Djafari-Bidgoli Z., Batooli H. (2009). GC/MS analysis and *in vitro* antioxidant activity of essential oil and methanol extracts of *Thymus caramanicus* Jalas and its main constituent carvacrol. Food Chem..

[5] Cosentino C., Tuberoso C.I.G., Pisan B., Satta M., Arzedi E., Palmas F. (1999). *In-*v*itro* antimicrobial activity and chemical composition of Sardinian *Thymus* essential oils. Lett. Appl. Microbiol..

[6] Lawrence B.M., Tucker A.O. (2002). The genus *Thymus* as a source of commercial products. Med. Arom. Plants – Ind. Prof..

[7] Ložiene K., Venskutonis P.R. (2005). Influence of environmental and genetic factors on the stability of essential oil composition of *Thymus pulegioides*. Biochem.Syst. Ecol..

[8] Dorman H.J., Deans S.G. (2000). Antimicrobial agents from plants: antibacterial activity of plant volatile oils. J. Appl. Microbiol..

[9] Jalas J., Tutin T.G., Heywood V.H., Burges N.A., Moore D.M., Valentine D.H., Walters S.M., Webb D.A. (1972). Flora Europaea.

[10] Gortzi O., Lalas S., Chinou I., Tsaknis J. (2006). Reevaluation of antimicrobial and antioxidant activity of *Thymus* spp. extracts before and after encapsulation in liposomes. J. Food Prot..

[11] Chorianopoulos N., Kalpoutzakis E., Aligiannis N., Mitaku S., Nychas G.-J., Haroutounian S. A. (2004). Essential oils of *Satureja*, *Origanum*, and *Thymus* species: chemical composition and antibacterial activities against foodborne pathogens. J. Agric. Food Chem..

[12] Grujic-Jovanovic S., Marin P.D., Dzamic A., Ristic M. (2009). Essential oil composition of *Thymus longicaulis* from Serbia. Chem. Nat. Comp..

[13] Baser K.H.C., Koyuncu M. (1994). Composition of the essential oils of two varieties of *Thymus longicaulis* C. Presl. subsp. *chaubardii* (Boiss. et Heldr. ex Reichb. fil.) Jalas. J. Essent. Oil Res..

[14] Baser K.H.C., Ozek T., Kirimer N., Tumen G. (1993). The occurrence of three chemotypes of *Thymus longicaulis* C. Presl. subsp. *longicaulis* in the same population. J. Essent. Oil Res..

[15] Baser K.H.C., Ozek T., Kurkcuoglu M., Tumen G. (1992). Composition of the essential oil of *Thymus logicaulis* C. Presl. var. *subisophyllus* (Borbas) Jalas from Turkey. J. Essent. Oil Res..

[16] Bellomaria B., Hruska K., Valentini G. (1981). Essential oils of *Thymus longicaulis* C. Presl from different localities of central Italy. Giorn. Bot. Ital..

[17] Mockute D., Bernotiene G. (2000). The α-terpenyl acetate chemotype of essential oil of *Thymus pulegioides* L. Biochem. Syst. Ecol..

[18] Pinto E., Pina-Vaz C., Salgueiro L., Gonçalves M.J., Costa-de-Oliveira S., Cavaleiro C., Palmeira A., Rodrigues A., Martinez-de-Oliveira J. (2006). Antifungal activity of the essential oil of *Thymus pulegioides* on *Candida*, *Aspergillus* and dermatophyte species. J. Med. Microbiol..

[19] Senatore F. (1996). Influence of harvesting time on yield and composition of the essential oil of a Thyme (*Thymus pulegioides* L.) growing wild in Campania (Southern Italy). J. Agric. Food Chem..

[20] De Feo V., Bruno M., Tahiri B., Napolitano F., Senatore F. (2003). Chemical composition and antibacterial activity of essential oils from *Thymus spinulosus* Ten. (Lamiaceae). J. Agric. Food Chem..

[21] De Martino L., De Feo V., Formisano C., Mignola E., Senatore F. (2009). Chemical composition and antimicrobial activity of the essential oils from three chemotypes of *Origanum*
*vulgare* L. ssp. *hirtum* (Link) Ietswaart growing wild in Campania (Southern Italy). Molecules.

[22] Azaz A.D., Irtem H.A., Kurkcuoglu M., Baser K.H.C. (2004). Composition and the *in vitro* antimicrobial activities of the essential oils of some *Thymus* species. J. Bioscience.

[23] Tzakou O., Verykokidou E., Roussis V., Chinou I (1998). Chemical composition and antibacterial properties of *Thymus longicaulis* subsp. *chaubardii* oils: Three chemotypes in the same population. J. Essent. Oil Res..

[24] Ložiene K., Vaiciuniene J., Venskutonis P.R. (2003). Chemical composition of the essential oil of different varieties of thyme (*Thymus pulegioides*) growing wild in Lithuania. Biochem.Syst. Ecol..

[25] Bauer A.W., Kirby W.M.M., Sherris J.C., Turck M. (1966). Antibiotic susceptibility testing by a standardized single disk method. Am. J. Clin. Pathol..

[26] Nedorostova L., Kloucek P., Kokoska L., Stolcova M., Pulkrabek J. (2009). Antimicrobial properties of selected essential oils in vapour phase against foodborne bacteria. Food Control.

[27] Ložiene K., Venskutonis P.R., Šipailienė A. (2007). Radical scavenging and antibacterial properties of the extracts from different *Thymus pulegioides* L. chemotypes. Food Chem..

[28] Valero M., Giner M.J. (2006). Effects of antimicrobial components of essential oils on growth of *Bacillus cereus* INRA L2104 in and the sensory qualities of carrot broth. Int. J. Food Microbiol..

[29] Patakova D., Chladek M. (1974). Antibacterial activity of thyme and wild thyme oils. Pharmazie.

[30] Pioro-Jabrucka E., Suchorska-Tropilo K., Rzewuska M. (2007). Antibacterial activity of the essential oil of common thyme (*Thymus pulegioides* L.) and wild thyme (*Thymus serpyllum* L.). Herba Pol..

[31] Sokovic M., Griensven L.J.L.D. (2006). Antimicrobial activity of essential oils and their components against the three major pathogens of the cultivated button mushroom, *Agaricus bisporus*. EurJ. Plant Pathol..

[32] Jennings W., Shibamoto T. (1980). Qualitative Analysis of Flavour and Fragrance Volatiles by Glass Capillary Gas Chromatography.

[33] Davies N.W. (1990). Gas chromatographic retention indices of monoterpenes and sesquiterpenes on methyl silicone and Carbowax 20M phases. J. Chromatogr..

[34] Adams R.P. (2007). Identification of Essential Oil Components by Gas Chromatography/Mass Spectrometry.

